# Retrospective Evaluation of End-Diastolic Forward Flow and Restrictive Physiology in One Hundred and Sixty-Four Dogs with Pulmonary Stenosis

**DOI:** 10.3390/vetsci12020152

**Published:** 2025-02-11

**Authors:** Elisabetta Boz, Cesara Sofia Pergamo, Stefania Signorelli, Viviana Forti, Claudio Maria Bussadori

**Affiliations:** Clinica Veterinaria Gran Sasso, 20131 Milan, Italy; cesarasofiapergamo@clinicaveterinariagransasso.it (C.S.P.); stefaniasignorelli@clinicaveterinariagransasso.it (S.S.); vivianaforti@clinicaveterinariagransasso.it (V.F.); claudiobussadori@clinicaveterinariagransasso.it (C.M.B.)

**Keywords:** right ventricle, echocardiography, restrictive physiology, pulmonary stenosis, diastolic funtion

## Abstract

The study of the function of the right ventricle (RV) is a topic of great interest in the scientific community, and some studies have evaluated parameters of the right ventricular systolic function and have correlated them to various RV pathologies and possible clinical findings. Less information was obtained on the right ventricular diastolic function. In veterinary medicine, the characteristics of the restrictive RV and the presence of end-diastolic forward flow (EDFF) in patients with pulmonary stenosis have not yet been described. This type of flow is an antegrade flow that is observed with the Doppler study of the pulmonary artery and occurs at the end of the diastolic phase. Pulmonary valve stenosis (PVS) is one of the most common congenital heart diseases in dogs. The echocardiographic parameter that is mainly taken as a reference for evaluating the severity of the disease is the peak gradient of the pulmonary antegrade flow. For our retrospective study, we consider a total of 164 dogs with PVS divided into 149 dogs with type A and 15 type B, seen at a reference veterinary clinic from 2020 to 2024. All these dogs underwent echocardiographic examinations to observe the morphological characteristics of the PVS and consider the presence or absence of restrictive RV with EDFF. An independent sample *t*-test analysis revealed that the severity of PVS is greater in dogs with EDFF, both in the group of patients with type A stenosis and in patients with type B stenosis. This study suggests that RV restrictive physiology is common in dogs with severe PVS.

## Simple Summary

Pulmonary stenosis is one of the most common congenital cardiac pathologies in dogs. This determines an increase in afterload with consequent right ventricular remodeling with the development of concentric hypertrophy. This pathophysiological mechanism is linked to a reduction in right ventricular diastolic function, which takes on a restrictive aspect with the presence of an anomalous antegrade flow in the late diastolic phase. This study highlights how the restrictiveness of the right ventricle is linked to the presence of a severe pathological condition and is found in patients who present severe right ventricular hypertrophy associated with greater severity of the degree of stenosis with high values of velocity and transvalvular pulmonary gradient.

## 1. Introduction

Pulmonary stenosis (PS) is one of the most common congenital heart diseases in dogs [1,2,3]. The pathology’s degree of severity is identified in the peak velocity of the pulmonary antegrade flow and in the transpulmonary gradient [4,5,6]. Outcome data from retrospective studies identify the presence of clinical signs such as syncope, exercise intolerance or heart failure, tricuspid regurgitation, high values of pulmonary peak gradient, and annular hypoplasia as adverse to patient survival; in these patients, the pulmonary valvuloplasty procedure is indicated [7,8,9].

The study of the right ventricle (RV) function is a topic of great interest in human and veterinary medicine. The RV shows different alterations depending on the hemodynamic stress to which it is subjected. During pulmonary stenosis, there is a condition of increased afterload that determines concentric right ventricular hypertrophy. The RV of younger patients appears to exhibit better adaptation to increased afterload. The myocardial tissue responds to this hemodynamic condition with the hypertrophy of cardiomyocytes and the increase in the number of capillaries in the young subject compared to the elderly [10,11]. Despite this ability, it is still recommended to intervene early in children to avoid the onset of right ventricular dilation and dysfunction [12]. The study of right ventricular function in dogs with pulmonary stenosis mainly considers systolic function parameters [13,14,15]. The diastolic function of these subjects has been less investigated. In human medicine, the restrictive pattern of the RV with the presence of end diastolic forward flow (EDFF) helps to formulate the long-term outcome in subjects undergoing repair procedures for tetralogy of Fallot [16,17]. The presence of RV restrictive pattern in adult subjects with PVS and its clinical implication was also studied [18]. The objective of this study is to investigate the presence of a restrictive RV physiology in dogs with PVS and observe a possible correlation with the severity of pathology.

## 2. Materials and Methods

All medical records of dogs affected by PVS that arrived at a veterinary cardiological reference center from January 2020 to August 2024 were retrospectively evaluated.

All dogs affected by PVS were included in this study and a complete transthoracic echocardiographic examination (TTE) was performed with a study of the right outflow tract and a Doppler study of the pulmonary antegrade flow. Exclusion criteria were the presence of other congenital/acquired cardiac or systemic disorders.

In all selected patients, medical history, physical examination, body weight at presentation, non-invasive systemic blood pressure (oscillometric method), dorsoventral and lateral thoracic radiographs were obtained, and standard twelve-lead electrocardiograms were registered. The TTE exams were performed by cardiology residents or a cardiology diplomate (C.B.), and all exams were reviewed by an ECVIM board-certified cardiologist (C.B.). The bidimensional (B-mode), monodimensional (M-mode), and spectral and color-flow Doppler images were obtained using Philips Epiq 7 [Philips Epiq 7C, Philips SpA Healthcare, Monza (MB), Italy], according to published recommendations [19].

Gender and age of the patients were considered to evaluate any correlation with the presence and absence of EDFF. We considered the following two different groups of patients based on age: in the first group, patients were less than one year old, while in the second group, patients were more than one year old.

**Figure 1:**
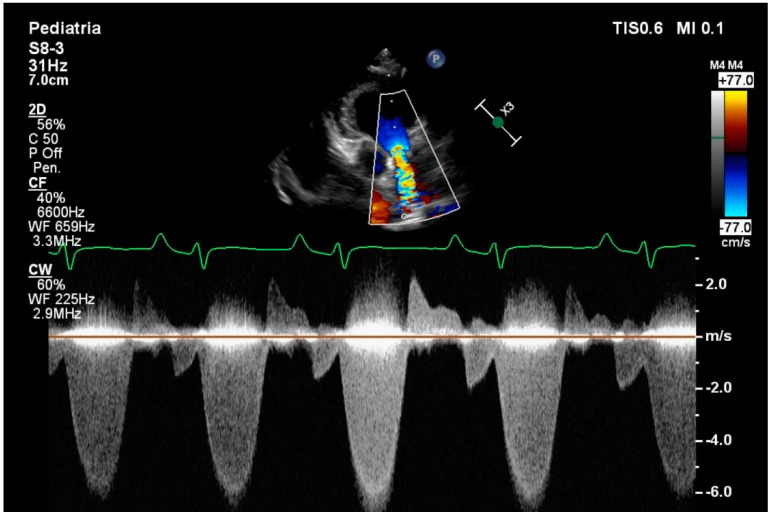
Evaluation of anterograde pulmonary flow of a patient with type B pulmonary stenosis with continuous−wave Doppler. Each flow in the systolic phase is preceded by a smaller flow that corresponds to the end diastolic phase, which is called EDFF. This anomalous flow is present in more than three consecutive beats.

The diagnosis of PVS was established when a mild, moderate, or severe narrowing of the pulmonary valve or of the RV outflow tract had been identified. This anomaly determines the acceleration of the pulmonary antegrade flow and increases the gradient across the pulmonary valve [4]. The evaluation of the RV outflow tract and of the pulmonary valve was obtained from the right parasternal short axis view and left parasternal short axis view [19]. With these projections, measurements of the diameter of the pulmonary annulus were also carried out, necessary to classify the type of stenosis distinguishing type A, type B, and hourglass morphology [4]. The M-mode measurements were obtained from the two-dimensional images of the largest transversal diameter of the LV on the right parasternal short axis view, at the level between papillary muscles and the mitral valve. In this projection, the free wall thickness of the right ventricle at the end of diastole (RVFWd) was measured at the level of the midventricle, excluding the pericardium and including the endocardium, as previously described [20]. The RVFWd was indexed by body weight (BW; iRVFWd = cm/kg^0.250^). The TAPSE measurement was performed through the apical 4-chamber view, applying the M mode recordings of the lateral aspect of the tricuspid valve annulus [21]; also, this measure was indexed to BW using the previously published scaling exponent (iTAPSE in mm/kg^0.297^) [21]. The Doppler study of pulmonary antegrade flow was performed both with the right parasternal short axis projection and with the left parasternal short axis view [19]. In each patient, the mean and the peak velocity of the antegrade pulmonary flow were measured, and the transvalvular gradient was calculated. This parameter is used to classify the severity of the pathology. Through this Doppler study, it was also possible to identify the presence or absence of the EDFF. To be considered a restrictive condition, EDFF must be present in at least three consecutive beats [18] (Figure 1).

Any type of arrhythmia was recorded and evaluated through electrocardiographic examination.

## 3. Statistical Analysis

Statistical analysis was performed using IBM SPSS Statistics. Distribution of variables was tested for normality using the Shapiro–Wilk test at the α = 0.05 level. As central position values, we considered the mean with standard deviation (St. Dev.) and interquartile range (IQR) as their respective dispersion indices; confidence intervals (CI) were calculated at 95% confidence. Normally distributed data were compared by the two-sided Student’s *t*-test for independent or paired samples as appropriate; non-normally distributed data were compared by the Mann–Whitney U test for independent samples. The chi-square test was used for the comparison of categorical variables. The value of *p* < 0.05 was considered significant.

## 4. Results

For this study, 202 dogs with pulmonary stenosis were considered and were observed from January 2020 to August 2024. For each patient the presence or absence of EDFF was observed. Of the total group, 182 cases were classified as type A pulmonary stenosis and 20 as type B pulmonary stenosis. From both groups, patients who presented congenital heart diseases associated with pulmonary stenosis were not taken into consideration. Therefore, the total number of subjects considered was 164 dogs, of which 149 were affected by type A pulmonary stenosis and 15 with type B stenosis (Table 1) In the group of patients suffering from type A pulmonary stenosis, 83.29% had a stenosis condition classified as severe, with a peak transvalvular gradient greater than 80 mmHg, 14.12% had a moderate condition with a gradient between 50 and 80 mmHg, and 2.59% had a mild stenosis, with a gradient lower than 50 mmHg. In the group of dogs with type B stenosis, 92.70% of the cases were affected by the severe form, 7.30% of the cases were affected by the moderate form, while there were no subjects with the mild pathological form. In both groups, patients with type A PVS and patients with type B PVS, gender and age were taken into consideration to verify that there was no correlation between the parameters and the presence and absence of EDFF. In terms of age, two groups of patients were considered: those aged less than one year and those aged over one year.

In the group of type A pulmonary stenosis without EDFF, 39.43% of patients were female and 60.56% were male, while the dogs with EDFF were 47.43% female and 52.56% were male. No significant difference was identified between the two groups. Regarding age, in the group of type A pulmonary stenosis without EDFF, 22.5% of patients were younger than one year old and 77.46% were older than one year old, while amongst the dogs with EDFF, 33.33% were younger than one year and 66.66% were older than one year old, without significant difference between groups. Dogs with type B pulmonary stenosis without EDFF were all females, while patients presenting with EDFF were 50% male and 50% female. The subjects with absence of EDFF were three, two of whom were less than one year old, and one was more than one year old. Regarding patients with type B stenosis and presence of EDFF, 58.33% were younger than one year and 41.66% were older. There were no significant differences between the two groups.

Table 2 summarizes the echocardiographic parameters considered to analyze the severity of the pathology and compares them in the group of patients who presented EDFF and in the patients who did not present EDFF. In the group of patients suffering from type A pulmonary stenosis, 78 (52.3%) had EDFF preceding pulmonary antegrade flow, and 71 (47.65%) did not have EDFF. A significant difference between the mean values of the two groups was observed in all parameters.

In the group of patients with type A pulmonary stenosis, 9.3% had an hourglass shape of the stenosis while 90.6% did not have an hourglass shape associated with the stenosis. The presence of a coronary anomaly was reported in only 3.3% of cases. No significance was identified with the presence of EDFF in type A PVS and the hourglass shape or the presence of coronary anomaly. In the group of patients with type B pulmonary stenosis, only three patients (20%) did not have EDFF, while twelve of them (80%) had the presence of EDFF. In this group of patients, a significant difference was highlighted in the mean values of the peak pulmonary velocity and the peak pulmonary gradient but not in the values relating to the thicknesses of the RVFWd and TAPSE (Table 3).

In patients with type B pulmonary stenosis, 46.6% had an hourglass shape while 53.3% did not; the association with coronary anomaly was only found in 13.3% of patients with type B pulmonary stenosis. No significance was observed between the presence of EDFF and the association of an hourglass form of stenosis or the presence of an anomalous coronary artery.

## 5. Discussion

In this study, we analyzed the presence of restrictive RV physiology in patients with PVS. We demonstrated that restrictive physiology is common in patients with moderate to severe type A (52.34%) and type B (80%) pulmonary stenosis. We also observed a correlation between the presence of EDFF in patients with type A PVS and greater RV hypertrophy and a reduction in TAPSE values. PVS is a common congenital heart disease in dogs. This represents a good sample for studying the right ventricular diastolic dysfunction and defining the clinical and echocardiographic characteristics that may have prognostic importance in these patients.

The echocardiographic study of right ventricular systolic function considers the use of TAPSE: measures of the maximum longitudinal distance the lateral tricuspid annulus travels toward the RV apex in systole. It is acquired from the left apical four-chamber view optimized for the RV with the M-mode cursor aligned through the lateral tricuspid annulus and originating from the RV apex. The cursor should be as parallel as possible with most of the RV free walls [21]. TAPSE reflects the longitudinal function of the right ventricle and is load dependent [22]; therefore, in pathologies that cause an increase in preload, such as atrial septal defects (ASDs) or tricuspid insufficiency (TI), an increase in this parameter is observed. Under conditions of increased afterload, an initial increase in TAPSE can be observed as a consequence of the Anrep effect [22]. Congenital pathologies that cause increased afterload, such as PVS, determine the development of circumferential fibers in the perinatal age with a prevalence of radial function over longitudinal function and a consequent reduction in TAPSE values while maintaining preserved systolic function [14,23]. Numerous studies have been published that report the repeatability of the measurement in dogs and identify reference values [20,21]. In these studies, a marked influence of the weight of the subjects on the TAPSE values was observed, and thus the use of body weight–specific reference values are advised. The variation of this parameter in the various pathologies affecting the right ventricle has been examined for patients such as boxers with arrhythmia [24], cats with hypertrophic cardiomyopathy [25,26], and even dogs with pulmonary stenosis [14]. In all these pathological forms, a reduction in the parameter correlated with a reduction in survival time was observed. In our study, reduced TAPSE and iTAPSE values are observed in all patients. In patients with type A pulmonary stenosis, a significant difference is observed between TAPSE and iTAPSE values between patients with EDFF and without EDFF, whereas the parameters are less reduced in the group without EDFF. This confirms what has been observed in previous studies [14]. In subjects with type B PVS, no significant difference is observed between the parameters of subjects with EDFF and those without EDFF. In this case, the sample is represented by a reduced number of subjects; furthermore, in these subjects, lower values of TAPSE and iTAPSE are highlighted even in subjects without EDFF, in line with the greater severity of the pathology linked to extreme morphological forms.

The other echocardiographic parameter that we considered is the thickness of the RVFWd taken in M-mode with the ultrasound beam directed towards the right parasternal two-dimensional short axis projection [20]. This parameter has also been used and defined by several studies in dogs and indexed to the weight of the patients. As identified in previous studies, an increase in RVFWd was observed in patients with pulmonary stenosis. This parameter is independently correlated to the severity of the pathology, considering the peak gradient [14]. In our patients, a significant difference is observed between RVFWd and iRVFWd in type A PS, where the thickness is greater in subjects with the presence of EDFF compared to those without EDFF (Table 2). This is correlated with what is observed from a physiopathological point of view in patients with this pathology and the consequent pressure overload that leads to concentric right ventricular hypertrophy. In patients with type B pulmonary stenosis, higher RVFWd and iRVFWd values are observed in the group without EDFF. However, the difference between the mean values is not significant, and we must always consider that in this case the sample is small (Table 3).

**Figure 2:**
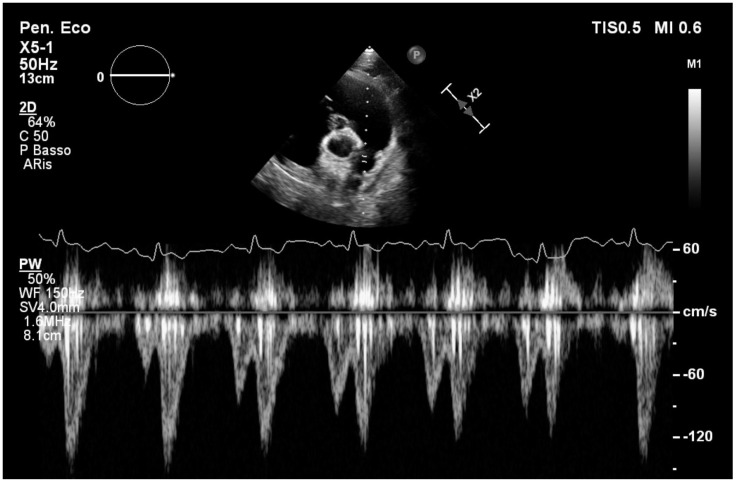
Pulsed-wave Doppler studies the pulmonary anterograde flow of a patient with arrhythmogenic cardiomyopathy. Each systolic flow is preceded by an anomalous antegrade flow during the end diastolic phase.

**Figure 3:**
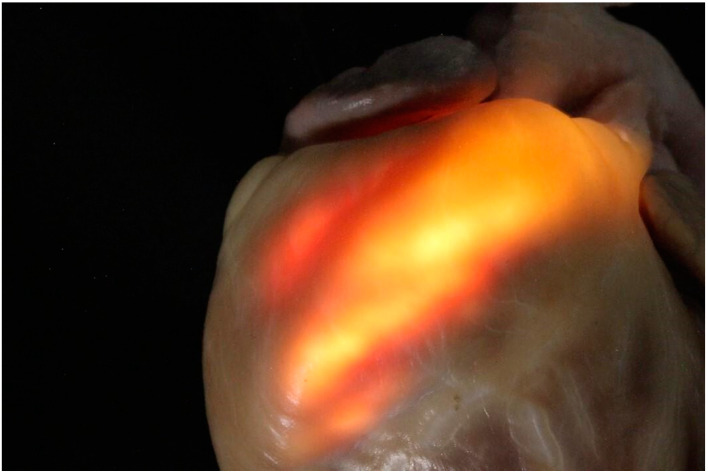
An anatomical pathological study of the right outflow tract of the same patient as in Figure 2. Translucency highlights the thinning of the wall linked to the arrhythmogenic cardiomyopathy.

The physiology of the restrictive RV is linked to its diastolic dysfunction [16,17,18], to the stiffening of the ventricular wall during the late diastole phase, which leads to the presence of an anomalous flow within the pulmonary artery during the atrial systole. This finding has been extensively studied in humans, particularly in patients with Tetralogy of Fallot undergoing a transannular patch surgical procedure. In the meta-analysis published by Van den Eynde J et al., in 2022, the results suggested that the presence of EDFF was related to right ventricular dilatation, right ventricular hypertrophy, and prolonged and severe pulmonary regurgitation [27,28]. Studies carried out on humans refer to two main pathological phenotypes in which EDFF can be observed: in the first, described by Cullen et al. and Gatzoulis et al., they refer to early-onset EDFF, the true restrictive right ventricle, with abnormal diastolic filling [16]. EDFF in these patients has its onset in the period around primary Tetralogy of Fallot repair. The second phenotype of EDFF is the late onset, “secondary”, one. This is a consequence of an enlargement of RV, usually related to longstanding pulmonary regurgitation and fibrosis [29]. What has also been reported in humans is that this anomalous flow can be influenced by the present respiratory phase; for this reason, the identification of a restrictive physiology must be correlated to the presence of EDFF throughout the respiratory cycle [29]. Other factors that can influence the presence of this anomalous flow are the absence of atrial systole and conditions that can reduce ventricular preload, linked to attenuation of the EDFF [30]. The presence of restrictive physiology of the right ventricle has been studied in adult patients with pulmonary stenosis, highlighting a correlation between its presence and the deterioration of the RV long axis function and a reduction in the patients exercise tolerance [18]. In dogs, the presence of restrictive physiology of the RV has been reported in dogs with PVS [31], but its correlation with a greater severity of the pathology has not yet been studied. In this case, the restrictiveness is linked to the presence of right ventricular hypertrophy given by the increase in afterload. This type of diastolic dysfunction seems related to the first phenotype described in humans, with a reduction in right ventricular filling also secondary to severe hypertrophy [32,33]. We observe the presence of EDFF also in subjects with chamber rigidity linked to right ventricular dilatation, such as in the English Bulldog with arrhythmogenic cardiomyopathy. In this condition, despite what is reported in the literature, where possible attenuation of EDFF is observed in the presence of tachyarrhythmias [30], a well-defined anterograde pulmonary flow in the end-diastolic phase was observed, probably related to the severe increase in right ventricular dimensions as well as to the presence of fibrosis, which conferred chamber stiffness. (Figure 2 and Figure 3).

## 6. Conclusions

Restrictive right ventricular physiology is common in patients with pulmonary stenosis. Its presence is correlated with a greater severity of the pathology and the presence of right ventricular hypertrophy, which causes right diastolic dysfunction. New investigations will have to be considered to identify this type of anomaly also in patients with other types of pathologies with greater involvement of the right heart and to identify whether this dysfunction is correlated with a negative prognostic condition of the patients.

## Figures and Tables

**Table 1:** Gender and age distribution in patients with type A pulmonary stenosis and type B pulmonary stenosis.

	Type A		Type B		Total	
	*n*	Perc.	*n*	Perc.	*n*	Perc.
Female	65	43.62%	9	60%	74	45.12%
Male	84	56.37%	6	40%	90	54.87%
<1 year old	38	25.50%	3	20%	41	25%
>1 year old	111	74.49%	12	80%	123	75%

**Table 2:** Echocardiographic parameters of patients affected by type A PS with and without EDFF.

	Type A with EDFF			Type A Without EDFF			*p*-Value
	Mean	St. Dev.	IQR	Mean	St. Dev.	IQR	
PV mean Velocity (m/s)	3.8	1.6	0.76	2.97	0.69	0.65	<0.001
PV mean Gradient (mmHg)	62.08	19.93	23.59	37.09	16.15	17	<0.001
PV peak Velocity (m/s)	5.65	0.86	1.22	4.15	0.96	1.18	<0.001
PV peak Gradient (mmHg)	129.73	41.91	49.50	73.5	32.69	44	<0.001
TAPSE (mm)	8.8	2.3	3	11.94	2.5	2.5	<0.001
iTAPSE	4.8	1.3	2	5.6	1.4	1.8	<0.001
RVFWd (cm)	0.75	0.25	0.32	0.58	0.20	0.27	<0.001
iRVFWd	0.44	0.15	0.18	0.30	0.10	0.12	<0.001

**Table 3:** Echocardiographic parameters of patients affected by Type B PS with and without EDFF.

	Type B with EDFF			Type B Without EDFF			*p*-Value
	Mean	St. Dev.	IQR	Mean	St. Dev.	IQR	
PV mean Velocity (m/s)	4.09	1.6	0.51	2.91	0.45	0.39	<0.001
PV mean Gradient (mmHg)	68.41	19.93	16	34.93	10.88	9.5	<0.001
PV peak Velocity (m/s)	5.7	0.63	0.79	4.4	0.57	1.15	0.003
PV peak Gradient (mmHg)	132.41	30.43	38.5	78.7	22.42	35.4	0.007
TAPSE (mm)	8.08	2.36	3.2	9.4	2.37	3.1	NS
iTAPSE	3.7	1.25	0.71	4.6	1.7	0.62	NS
RVFWd (cm)	0.99	0.25	0.19	1.1	0.20	0.15	NS
iRVFWd	0.53	0.28	0.15	0.55	0.33	0.18	NS

## Data Availability

Data is contained within the article.
